# Dual-FRET imaging of IP_3_ and Ca^2+^ revealed Ca^2+^-induced IP_3_ production maintains long lasting Ca^2+^ oscillations in fertilized mouse eggs

**DOI:** 10.1038/s41598-019-40931-w

**Published:** 2019-03-18

**Authors:** Toru Matsu-ura, Hideki Shirakawa, Kenichi G. N. Suzuki, Akitoshi Miyamoto, Kotomi Sugiura, Takayuki Michikawa, Akihiro Kusumi, Katsuhiko Mikoshiba

**Affiliations:** 1 Laboratory for Developmental Neurobiology, Center for Brain Sciences, RIKEN, 2-1 Hirosawa, Wako, Saitama 351-0198 Japan; 2 Department of Applied Physics and Chemistry, The University of Electro-Communications, Tokyo, 182-8585 Japan; 3 Center for Highly Advanced Integration of Nano and Life Sciences (G-CHAIN), Gifu University, 1-1 Yanagido, Gifu, 501-1193 Japan; 4 Laboratory of Single-Molecule Cell Biology, Kyoto University Graduate School of Biostudies, Konoe-cho, Sakyo-ku, Kyoto 606-8501 Japan; 5 Division of Mucosal Vaccines, International Research and Development Center for Mucosal Vaccine, The Institute of Medical Science, University of Tokyo, 4-6-1 Shirokanedai, Minato-ku, Tokyo, 108-8639 Japan; 6 Laboratory for Biotechnological Optics Research, Center for Advanced Photonics, RIKEN, 2-1 Hirosawa, Wako, Saitama 351-0198 Japan; 7 Okinawa Institute of Science and Technology Graduate University, 1919-1 Tancha, Onna, Okinawa 904-0495 Japan; 8 Department of Pharmacology, Keio University School of Medicine, 35 Shinanomachi, Shnjukuku, Tokyo 160-8582 Japan; 9 Shanghai Institute for Advanced Immunochemical Studies, ShanghaiTech University, Shanghai, 201210 China

## Abstract

In most species, fertilization induces Ca^2+^ transients in the egg. In mammals, the Ca^2+^ rises are triggered by phospholipase Cζ (PLCζ) released from the sperm; IP_3_ generated by PLCζ induces Ca^2+^ release from the intracellular Ca^2+^ store through IP_3_ receptor, termed IP_3_-induced Ca^2+^ release. Here, we developed new fluorescent IP_3_ sensors (IRIS-2s) with the wider dynamic range and higher sensitivity (Kd = 0.047–1.7 μM) than that we developed previously. IRIS-2s employed green fluorescent protein and Halo-protein conjugated with the tetramethylrhodamine ligand as fluorescence resonance energy transfer (FRET) donor and acceptor, respectively. For simultaneous imaging of Ca^2+^ and IP_3_, using IRIS-2s as the IP_3_ sensor, we developed a new single fluorophore Ca^2+^ sensor protein, DYC3.60. With IRIS-2s and DYC3.60, we found that, right after fertilization, IP_3_ concentration ([IP_3_]) starts to increase before the onset of the first Ca^2+^ wave. [IP_3_] stayed at the elevated level with small peaks followed after Ca^2+^ spikes through Ca^2+^ oscillations. We detected delays in the peak of [IP_3_] compared to the peak of each Ca^2+^ spike, suggesting that Ca^2+^-induced regenerative IP_3_ production through PLC produces small [IP_3_] rises to maintain [IP_3_] over the basal level, which results in long lasting Ca^2+^ oscillations in fertilized eggs.

## Introduction

In most species, rises in cytosolic Ca^2+^ concentration ([Ca^2+^]) trigger the egg-embryo transition. Unfertilized eggs, which are arrested at different stages of meiosis in different species, are “activated” and released from the arrest by fertilization^1,2^. In mammals, egg activation is triggered by a periodic series of Ca^2+^ transients, known as Ca^2+^ oscillations^3,4^. The response in mammalian eggs lasts for several hours and involves relatively low frequency, large amplitude Ca^2+^ increases^5^. The multiple increases in [Ca^2+^] are essential for completion of all the events of egg activation in mammals^5,6^.

The first Ca^2+^ transient occurs some minutes after sperm-egg fusion^7^. The Ca^2+^ oscillations in mammalian eggs appear to be a result of Ca^2+^ release via the inositol 1,4,5-trisphosphate (IP_3_) receptor/Ca^2+^ release channel (IP_3_R) located on the intracellular Ca^2+^ stores^8^. A sperm-specific phospholipase Cζ (PLCζ)^9^, which produces IP_3_ via hydrolysis of phosphatidyl 4,5-bisphosphate (PIP_2_), is reported as an egg-activating sperm factor^10^ in mammalian species. The microinjection of complementary RNA (cRNA) encoding PLCζ^11^ or recombinant PLCζ proteins^12^ into unfertilized mouse eggs triggers characteristic Ca^2+^ oscillations like those observed at fertilization. Sperm from transgenic mice with significantly reduced expression of PLCζ display a premature termination of Ca^2+^ oscillations following *in vitro* fertilization^13^. PLCζ shows extremely high Ca^2+^ sensitivity for its enzymatic activity compared with other PLC isoforms, with 70% maximal activity at 100 nM Ca^2+^ ^12^. Therefore, it has been considered that basal cytosolic Ca^2+^ in the fertilized egg can stimulate PLCζ to produce an amount of IP_3_ sufficient to trigger the initial release of Ca^2+^, which has not been confirmed experimentally, since single cell imaging using fluorescent IP_3_ indicators, such as green fluorescent protein (GFP)-fused to pleckstrin homology domain (GFP-PHD)^14^ and fretino-2^15^, failed to clearly detected IP_3_ concentration ([IP_3_]) changes evoked in fertilized mouse eggs^16,17^.

Because all PLC isoforms including PLCζ are activated by Ca^2+^ ^12,18–20^, there will be further increase in IP_3_ production when [Ca^2+^] start to increase. This positive feedback has been proposed to play a central role for the generation of the upstroke of Ca^2+^ transients^21,22^. Except for PLCζ, members of each of the PLC families are expressed in eggs, and PLCβ1 is reported to contribute generation of Ca^2+^ transients^23^ and in theory any of these could be involved in modulating Ca^2+^ oscillations. On the other hand, the positive feedback regulation of Ca^2+^ acting directly on the IP_3_R has been proposed to drive regenerative Ca^2+^ increases^24^. Simultaneous detection of [Ca^2+^] and [IP_3_] is necessary to figure out the contributions of Ca^2+^-induced IP_3_ production from PLCs and Ca^2+^ release form IP_3_R for the generation of fertilization-induced Ca^2+^ transients.

In the present study, we developed novel fluorescent resonant energy transfer (FRET)-based IP_3_ sensor proteins, designated as IRIS-2s, to visualize IP_3_ dynamics in fertilized mouse eggs. The novel IP_3_ sensors possess an improved dynamic range compared with the previous sensor, IRIS-1^25^. A high IP_3_ binding affinity variant, IRIS-2.3, can be successfully used to monitor [IP_3_] changes naturally induced in fertilized mouse eggs. IRIS-2s contain enhanced green fluorescent protein (EGFP) and Halo-protein with tetramethylrhodamine (TMR) ligand as FRET donor and acceptor, respectively. To monitor [Ca^2+^] and [IP_3_] changes simultaneously, we also developed a new Ca^2+^ sensor protein, designated as DYC3.60, which has enhanced cyan fluorescent protein (ECFP) as a solo fluorophore. The pair of IRIS-2s and DYC3.60 contains a new set of fluorophores for dual-FRET imaging, and real time monitoring with IRIS-2s and DYC3.60 provide us insights into the mechanism underlying the generation of Ca^2+^ oscillations in mouse fertilized eggs.

## Results

### Construction of IRIS-2s

**Figure 1:**
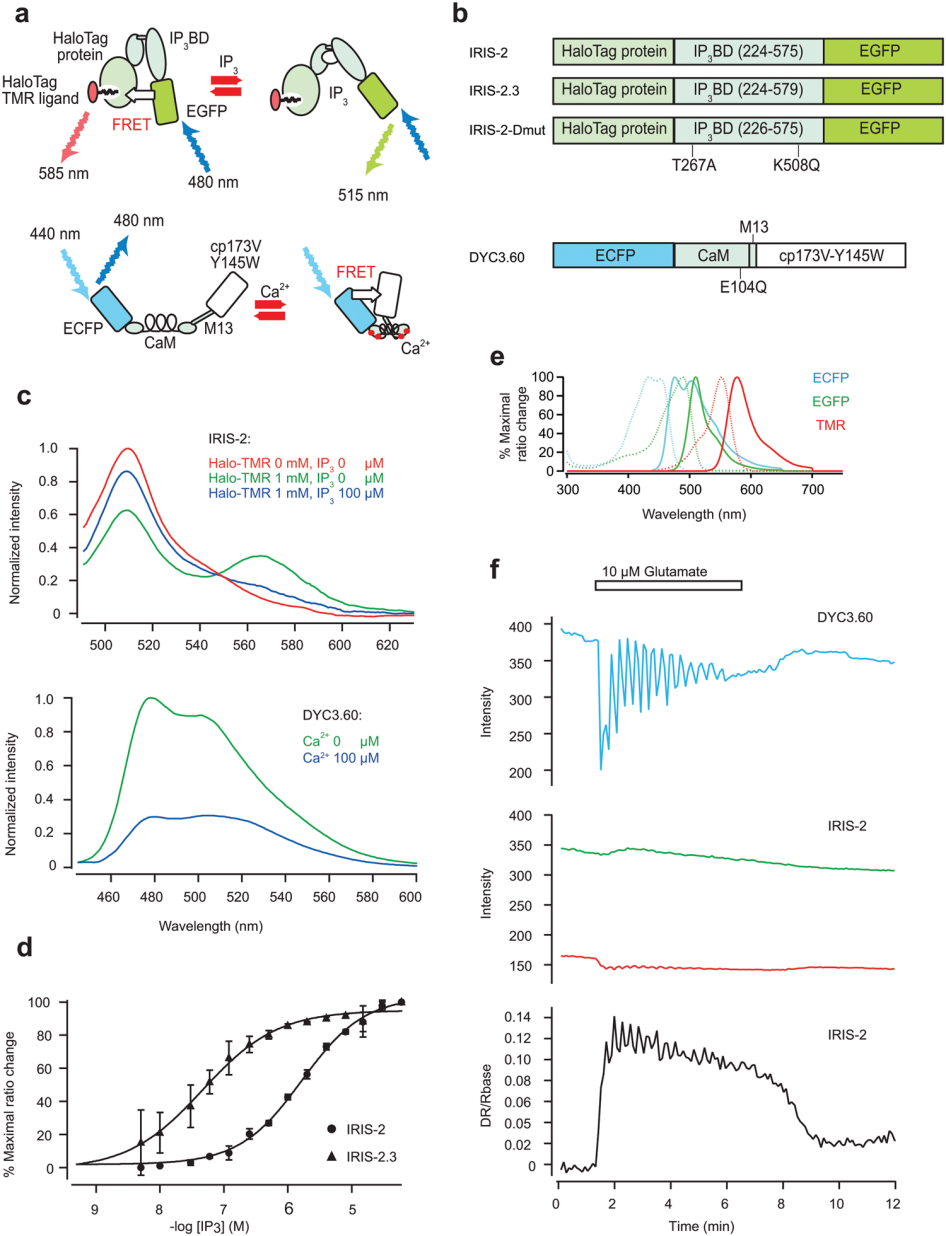
Construction and characterization of IRIS-2s and DYC3.60. (**a**) Schematic drawing of IRIS-2s and DYC3.60. IRIS-2s are composed of the IP_3_BD of mouse IP_3_R1, EGFP and HaloTag protein. HaloTag TMR ligand was used for the acceptor in IRIS-2s. DYC3.60 is composed of calmodulin (CaM), M13 peptide, ECFP and a non-fluorescent mutant of circular permutated Venus (cp173V-Y145W). (**b**) Domain structures of IRIS-2 proteins and DYC3.60. (**c**) Emission spectra of IRIS-2 (upper panel) and DYC3.60 (lower panel) excited at 480 and 440 nm, respectively. Spectra of purified IRIS-2 (red line), IRIS-2_TMR_ (in the presence of 1 μM HaloTag TMR ligand) (green line), and IRIS-2_TMR_ in the presence of 100 μM IP_3_ (blue line). Spectra of lysate of DYC3.60-expressing COS7 cells were measured with 0.1 mM of CaCl_2_ (blue line) or without CaCl_2_ (green line). (**d**) Apparent IP_3_ affinities of purified IRIS-2_TMR_ (circles) and IRIS-2.3_TMR_ (triangles). Data were obtained from three independent measurements. Error bars correspond to the SD. (**e**) Excitation (broken lines) and emission (continuous lines) spectra of ECFP, EGFP, and TMR. (**f**) Time courses of emission changes of DYC3.60 (blue line), EGFP (green line) and TMR (red) of IRIS-2_TMR_ in mGluR5 expressing HeLa cells stimulated with 10 μM glutamate (horizontal bars). The ratio of EGFP and TMR is drawn in the bottom panel (black line).

We constructed novel IP_3_ sensors composed of HaloTag protein (Promega), IP_3_ binding domain (IP_3_BD) of mouse IP_3_R1^25^, and mEGFP (upper panel in Fig. 1a). HaloTag protein is an engineered, catalytically inactive derivative of a hydrolase that forms a covalent bond with commercially available HaloTag ligands. We used HaloTag^*®*^ tetramethylrhodamine (TMR) ligand (Promega) as a FRET acceptor for mEGFP. Amino acid residues 224–575 and 224–579 of mouse IP_3_R1 were used for IRIS-2 and IRIS-2.3, respectively (upper panel in Fig. 1b), to manipulate the IP_3_ binding affinity of the sensors. We constructed IRIS-2-Dmut, in which two critical amino acid residues (Thr267 and Lys508) for IP_3_ binding have been replaced in IRIS-2, as a negative control^25^ (upper panel in Fig. 1b). The upper panel in Fig. 1c shows emission spectrum of IRIS-2 when excited at 480 nm. Purified IRIS-2 with addition of HaloTag TMR ligand (IRIS-2_TMR_) showed greater TMR emission (565 nm) and lesser EGFP emission (510 nm) (green line in Fig. 1c) compared with those of untreated IRIS-2 (red line in Fig. 1c), indicating that FRET between EGFP and TMR occurs in IRIS-2_TMR_. The addition of 100 μM IP_3_ increased the EGFP emission and decreased the TMR emission (blue line in Fig. 1c), indicating that the FRET efficiency of IRIS-2_TMR_ decreases upon IP_3_ binding (Fig. 1a). The relative change in the EGFP/TMR emission ratio of IRIS-2_TMR_ monitored with zero and 100 μM IP_3_ (155 ± 26%; n = 3) was three times larger than that of IRIS-1 (55.2 ± 2.7%; n = 3) (Fig. 1c and Supplementary Fig. 1). The high dynamic range achieved in IRIS-2 was preserved in IRIS-2.3 treated with HaloTag TMR ligand (IRIS-2.3_TMR_) (117 ± 3%; n = 3). Figure 1d shows the IP_3_ dependence of the emission ratio of IRIS-2_TMR_ and IRIS-2.3_TMR_. The Kd value of IRIS-2.3_TMR_ (0.047 ± 0.006 μM; n = 3; Fig. 1d) was 36-times smaller than that of IRIS-2_TMR_ (1.7 ± 0.2 μM; n = 3; Fig. 1d). The Kd value of IRIS-2_TMR_ was 3-times larger than that of IRIS-1 (0.55 ± 0.06 μM)^25^.

As a partner of IRIS-2s, we developed a FRET based Ca^2+^ indicator with single fluorophore to avoid fluorescent overlapping with IRIS-2s. We introduced a non-fluorescent mutation (Y145W^26^) into a yellow fluorescent protein, cp173Venus, of YC3.60^27^ (lower panels in Fig. 1a,b). The resultant protein have a fluorescent spectrum as same as ECFP, and addition of 100 μM Ca^2+^ decreased its emission by FRET quenching. The peak fluorescent amplitude was 71 ± 3% (n = 3) reduced after addition of Ca^2+^ in DYC3.60 (lower panel in Fig. 1c). Fluorescence from the three fluorophores used in IRIS-2s and DYC3.60 can be easily separated (Fig. 1e). Figure 1f shows time course changes of fluorescence from DYC3.60 and IRIS-2 in glutamate stimulated mGluR5-expressing HeLa cells. Less overlaps of excitation and emission spectra of IRIS-2 and DYC3.60 allowed dual-FRET imaging of Ca^2+^ and IP_3_ even without spectral unmixing^28^ (Fig. 1f).

### Characterization of IRIS-2s and DYC3.60 expressed in cultured mammalian cells

**Figure 2:**
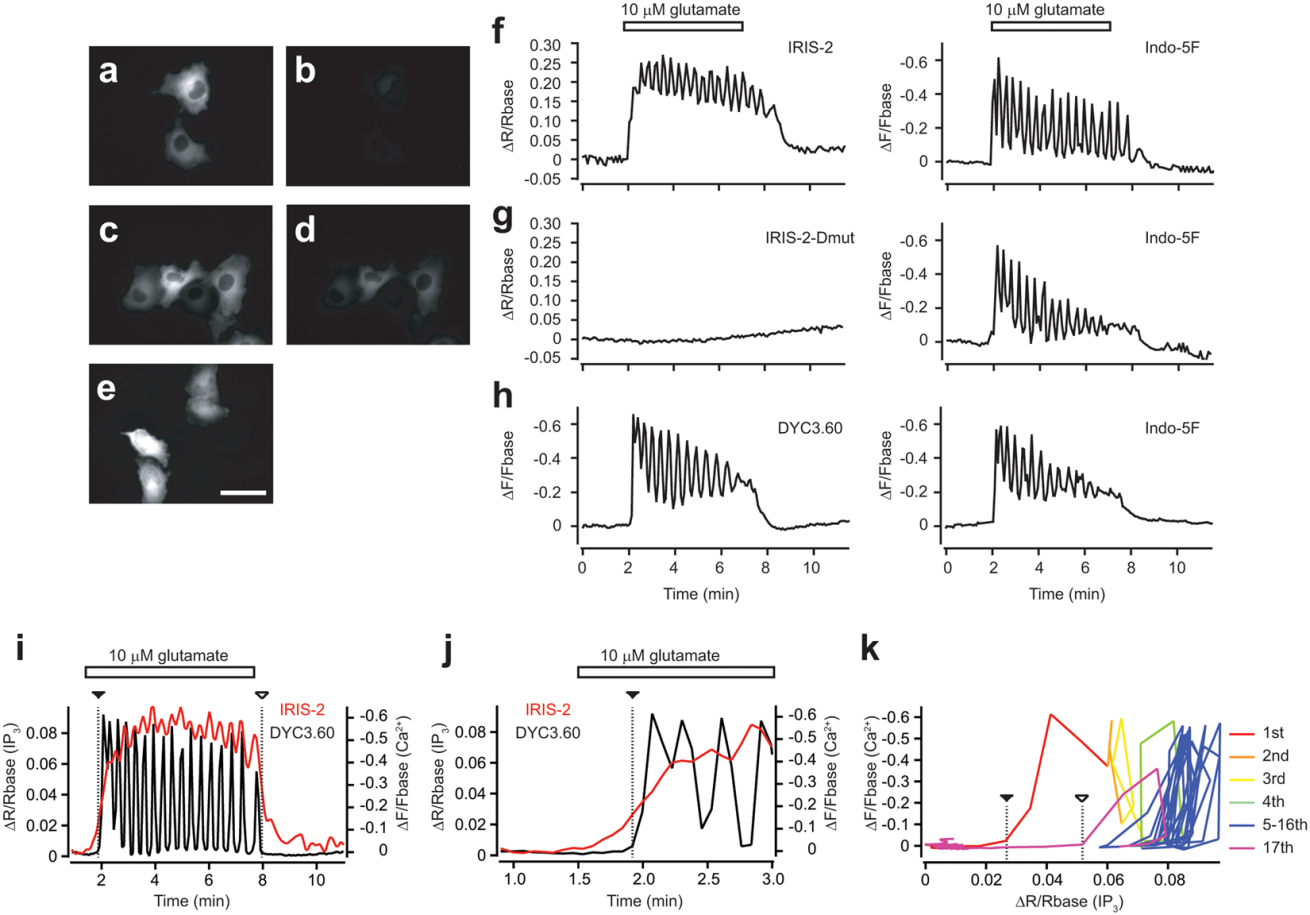
Effect of FRET sensors to [Ca^2+^] dynamics in HeLa cells. (**a**–**d**) Fluorescent images of IRIS-2 expressing cells without (**a**,**b**) and with HaloTag TMR staining (**c**,**d**). Bar, 50 μm. Emissions detected by filters for GFP are shown in (**a**,**c**). Emissions detected by filters for TMR are shown in (**b**,**d**). (**e**) A fluorescent image of DYC3.60 expressing cells. (**f**–**h**) Effect of FRET sensors to [Ca^2+^] dynamics in HeLa cells. Cells expressing IRIS-2 (**f**), IRIS-2-Dmut (**g**), or DYC3.60 (**h**) were stained with HaloTag TMR and Indo-5F. Left panels are time courses of emission ratio (**f,g**) or emission (**h**) changes of FRET sensors in mGluR5 expressing HeLa cells stimulated with 10 μM glutamate (horizontal bars). Right panels are time courses of emission changes of Indo-5F in the same cells of left panels. (**i**–**k**) [IP_3_] and [Ca^2+^] changes were imaged with the dual FRET probes. Initiation of the first Ca^2+^ spike and the termination of Ca^2+^ oscillations were marked with dashed line with closed and open triangles, respectively. (**j**) [IP_3_] and [Ca^2+^] changes around the rise of first Ca^2+^ spike. (**k**) A phase plane trajectory is drawn with [IP_3_] and [Ca^2+^] imaging data.

IRIS-2_TMR_ and DYC3.60 were uniformly distributed within the cytosol when expressed in HeLa cells (Fig. 2a–e). Halo-TMR staining increased fluorescent signal detected by a 573–613-nm emission filter (Fig. 2b,d). The frequency of Ca^2+^ oscillations monitored with Indo-5F in mGluR5-expressing HeLa cells stimulated with 10 μM glutamate were not significantly different among IRIS-2-, IRIS-2-Dmut-, and DYC3.60-expressing cells (50 ± 16 mHz for IRIS-2, n = 9; 55 ± 6 mHz for IRIS-2-Dmut, n = 4; 47 ± 10 mHz, n = 6 for DYC3.60) (Fig. 2f–h). IRIS-2_TMR_ signals did not return to its basal level during the intervals between Ca^2+^ transients, and its fluctuation was synchronous with Ca^2+^ oscillations (Fig. 2f). These characteristic IP_3_ dynamics monitored with IRIS-2_TMR_ in HeLa cells are almost same as those recorded with other FRET-based IP_3_ sensors^15,25,29,30^.

### Initiation, maintenance, and termination of Ca^2+^ oscillations in HeLa cells

Figure 2i–k show imaging data of IRIS-2 and DYC3.60 in mGluR5-expressing HeLa cells. The dual FRET imaging clearly showed [IP_3_] increase precedes [Ca^2+^] rise as same as our previous report with IRIS-1^25^ (Fig. 2i,j). Figure 2k shows a phase plane trajectory of [IP_3_] and [Ca^2+^] imaging data. [IP_3_] gradually increased from 1st to 4th Ca^2+^ spikes, and then, repeated Ca^2+^ spikes occurred in the certain range of [IP_3_] (Fig. 2k). In the range of [IP_3_], the trajectory cycled at almost the same orbit, suggesting that the trajectory is in a limit cycle (Fig. 2k). After termination of agonist stimulation, [IP_3_] decreased below the range of limit cycle maintenance, which resulted in the termination of Ca^2+^ oscillations (Fig. 2i,k). In the initial phase of Ca^2+^ oscillations, [IP_3_] increase precedes Ca^2+^ spikes (Fig. 2k), suggesting that [IP_3_] increases induce Ca^2+^ spikes. In the limit cycle phase, Ca^2+^ spikes occur without marked [IP_3_] increases (Fig. 2k), suggesting that Ca^2+^ induced positive and negative feedbacks to IP_3_R autonomously induce Ca^2+^ spikes^24^. Ca^2+^ oscillations last as long as [IP_3_] maintained in the range of limit cycle. Termination of agonist stimulation induces [IP_3_] decrease below to the range maintaining the limit cycle. [IP_3_] necessary to induce Ca^2+^ spike should be different at the initial state and later state of Ca^2+^ oscillations because Ca^2+^ directly or indirectly inactivates IP_3_R^28,31^. Thus, Ca^2+^ disappears even [IP_3_] above the basal level at the termination of Ca^2+^ oscillations (Fig. 2i).

### Characterization of IRIS-2 in UV-uncaging experiments

Next, we checked the compatibility of IP_3_ sensors with UV-uncaging. Caged-compounds are light-sensitive probes that functionally encapsulated biomolecules in an inactive form. The active compounds can be released from caged-compounds with UV light in most of caged-compounds. IRIS-1 or IRIS-2 were expressed in HeLa cells and irradiated by UV pulses (Supplementary Fig. 2a,b). We found UV irradiation caused temporal reduction of fluorescence of both ECFP and Venus in IRIS-1 expressing cells (Supplementary Fig. 2a). Because of the difference of the signal reduction between those fluorescent proteins, the fluorescent ratio of IRIS-1 was significantly reduced (−1.9 ± 0.7%, n = 22). In contrast, the fluorescent signals from EGFP and HaloTag-TMR were stable after the UV irradiation (Supplementary Fig. 2b), which resulted in successful detection of [IP_3_] changes after UV-uncaging of caged-IP_3_ (Supplementary Fig. 2c).

### Detection of IP_3_ concentration changes in fertilized mouse eggs

**Figure 3:**
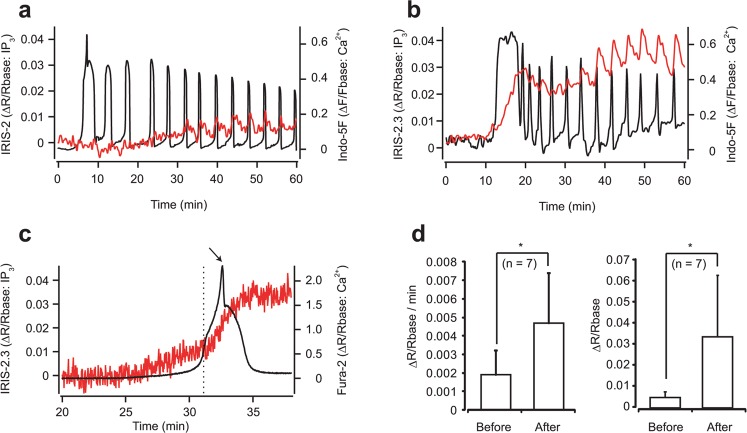
IRIS-2 _TMR_ and IRIS-2.3 _TMR_ signals in fertilized mouse eggs. (**a**,**b**) [IP_3_] and [Ca^2+^] dynamics detected by IRIS-2s and Indo-5F. Normalized emission ratio changes (∆R/Rbase) of IRIS-2_TMR_ (**a**) and IRIS-2.3_TMR_ (**b**) are plotted with red lines. [Ca^2+^] changes detected with Indo-5F are shown with black lines (**a**,**b**). Sperm was added at time zero (**a**,**b**). Fluorescent images were acquired each 4 sec in (**a**) and each 10 sec in (**b**). (**c**) Time courses of emission ratio changes of Fura-2 (black) and IRIS-2.3_TMR_ (red) at a first Ca^2+^ spike after fertilization. The average values from whole egg were plotted against time. A peak of the first Ca^2+^ spike is shown by an arrow. The time point of the change in the rate of rise in the IRIS-2.3_TMR_ signal is shown by a vertical broken line. (**d**) Left panel shows the rates of [IP_3_] increases before and after the shoulder point of the first Ca^2+^ spike. Right panel shows peak [IP_3_] change before [Ca^2+^] rise and first [IP_3_] peak after [Ca^2+^] rise. n = 7. *p < 0.05, Student’s t-test.

To detect IP_3_ dynamics in fertilized mouse eggs, IRIS-1, IRIS-2, or IRIS-2.3 was expressed in eggs by cRNA injection. For the simultaneous monitoring of [Ca^2+^] changes, we first used Indo-5F as a Ca^2+^ indicator according to the method described previously^25^. As shown in Supplementary Figure 3, we did not detect any changes of IRIS-1 signals in fertilized eggs. Not only the fails of the detection of IP_3_ changes, it was difficult to detect [Ca^2+^] changes after addition of sperm into the culturing media. Even in the experiments with successful detection of fertilization-induced [Ca^2+^] changes, the number of Ca^2+^ transients was less compared to IRIS-2-Dmut (number of Ca^2+^ spikes during 30 min after the first Ca^2+^ spikes: IRIS-1: 1.91 ± 0.13 (n = 3); IRIS-1-Dmut: 3.75 ± 0.5 (n = 4); p = 0.008, Student’s t-test), suggesting that IRIS-1 works as a significant IP_3_ buffer. We also tested IRIS-2 expressing eggs for *in-vitro* fertilization assay and found IRIS-2 expressing eggs had normal Ca^2+^ spikes after fertilization (Fig. 3a). However, it was also hard to detect clear increases in FRET signals in IRIS-2-expressing eggs during the first Ca^2+^ transient evoked after fertilization, while small repetitive transients of IRIS-2_TMR_ signals synchronous with Ca^2+^ oscillations were observed approximately 30 min after the onset of the first Ca^2+^ transient (Fig. 3a). On the other hand, we clearly detected IP_3_ increases during the all Ca^2+^ transients, including the first Ca^2+^ transient, in IRIS-2.3-expressing eggs (Fig. 3b). During the first large Ca^2+^ transient, [IP_3_] continues to increase, and all the following Ca^2+^ transients accompanied with a rapid increase and a following slow decline on the elevated level of [IP_3_] (Fig. 3b). Three independent experimental results of [IP_3_] and [Ca^2+^] imaging with IRIS-2.3 and Indo-5F at the onset of first Ca^2+^ spike were shown in Supplementary Figure 4. We did not find significant difference of numbers of Ca^2+^ spikes during 30 min after 1st Ca^2+^ spike between IRIS-2 and IRIS-2.3 expressing eggs (IRIS-2: 5.17 ± 1.72, n = 6; IRIS-2.3: 6.33 ± 5.72, n = 9; p = 0.58, student’s t-test).

### Initiation of [IP_3_] and [Ca^2+^] changes

Next, we investigated the temporal order of the onset of increase between [IP_3_] changes and [Ca^2+^] changes during the first Ca^2+^ transient evoked after fertilization. To detect the initial [Ca^2+^] changes experimentally, we used Fura-2, whose affinity is higher than that of Indo-5F (Fura-2: Kd = 135 nM; Indo-5F: Kd = 470 nM), as a Ca^2+^ indicator to detect the timing of the onset of the first Ca^2+^ transient as precise as possible. As shown in Fig. 3c, [IP_3_] rise preceded the onset of the initial step of the first Ca^2+^ transient for 2.7 ± 2.4 min in 11 of 13 eggs. The initial [IP_3_] increase should initiate Ca^2+^ release from IP_3_R. IP_3_ and Ca^2+^ are the co-agonist of IP_3_R, and open probability of IP_3_R markedly increase with Ca^2+^ in the presence of IP_3_^32^. Thus, Ca^2+^-induced Ca^2+^ release (CICR) from IP_3_R should have major role to produce initial Ca^2+^ spike. The first Ca^2+^ transient observed in IRIS-2.3-expressing eggs was composed of two steps separated by a shoulder point (dashed line in Fig. 3c) as reported previously^33^. The peak amplitude and the rising speed of [IP_3_] increased after the shoulder point of the first Ca^2+^ transient (Fig. 3c,d), suggesting acceleration of IP_3_ production via Ca^2+^-induced activation of PLC isozymes.

### Positive feedback loop to produce rising phase of Ca^2+^ spikes

**Figure 4:**
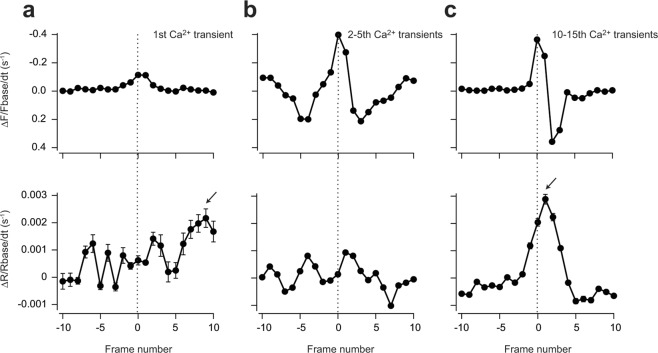
Rate of [Ca^2+^] and [IP_3_] changes at each Ca^2+^ spike. Rate of [Ca^2+^] and [IP_3_] changes are shown as differentiated signals of Indo-5F (Em. 460–510 nm) (upper panel) and IRIS-2.3_TMR_ (lower panel) aligned by the time when the differentiated Indo-5F signal was at its maximum (frame 0, broken line) during first Ca^2+^ transients after fertilization (n = 9) (**a**), from 2nd to 5th Ca^2+^ transients (n = 20) (**b**) and from 10th to 15th Ca^2+^ transients (n = 20) (**c**). Error bars correspond to the SD. Arrowheads indicate the peak of the rate of [IP_3_] rise. Broken vertical lines indicate the peaks of differentiated Indo-5F signals.

Each Ca^2+^ spike of Ca^2+^ oscillations usually form as a result of an initial slow pacemaker rise in [Ca^2+^] followed by a rapid rise in [Ca^2+^]^34–36^. The accelerated rise of [Ca^2+^] is suggested that a regenerative process is involved in the generation of the abrupt upstroke^35^. Such regenerative processes require a positive-feedback element^22^, and CICR from IP_3_R and Ca^2+^-induced IP_3_ production through PLC have been proposed as candidates of the positive feedback element. In the previous study, we compared rate of [IP_3_] and [Ca^2+^] rises at the onset of Ca^2+^ spikes and found each Ca^2+^ spike is not accompanied by acceleration in the rate of increase in IP_3_ in HeLa cells^25^. As same as HeLa cells, if the regenerative IP_3_ production mediated by PLC activated by cytosolic Ca^2+^ drives the rising phase of Ca^2+^ spikes, the rate of [IP_3_] rise should accelerate when the rate of [Ca^2+^] rise accelerate. To test this possibility, the fluorescent signals of both Indo-5F and IRIS-2.3_TMR_ were differentiated and aligned at the time when the rate of [Ca^2+^] rise reached its maximum (Fig. 4). In the early phase (from first to 5th transients) of fertilization-induced Ca^2+^ oscillations, the amplitudes of IP_3_ fluctuations were relatively small (Fig. 3b), and the rate of [IP_3_] rise did not increase during the rising phase of the Ca^2+^ transients, as found in cultured HeLa cells^25^ (Fig. 4a,b). The amplitudes of IP_3_ fluctuations were gradually increased during the later phase of Ca^2+^ oscillations (Fig. 3a,b), and contrary to the early phase, the onset of the rate of [IP_3_] rise precedes that of [Ca^2+^] (Fig. 4c). The result suggests that Ca^2+^-induced IP_3_ production through PLC may work as a part of the positive feedback loop to produce abrupt [Ca^2+^] rise at Ca^2+^ spikes in later phase of Ca^2+^ oscillations. However, the peak of the rate of [IP_3_] rise always delayed from that of [Ca^2+^] (Fig. 4c), suggesting that CICR from IP_3_R has major role to produce the rising phase of Ca^2+^ spikes and [IP_3_] rises.

[IP_3_] stayed at the elevated level and did not return to the basal level through Ca^2+^ oscillations (Fig. 3b).

### Dual-FRET imaging of [IP_3_] and [Ca^2+^] in fertilized mouse eggs

**Figure 5:**
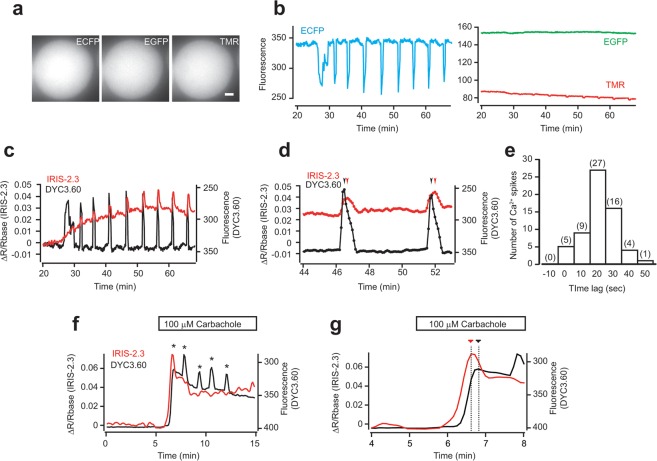
Delayed IP_3_ pulses during Ca^2+^ oscillations in fertilized mouse eggs visualized by dual-FRET sensors. (**a**) Fluorescence images of DYC3.60 (ECFP) and IRIS-2.3_TMR_ (EGFP and TMR) in a single mouse egg. DYC3.60 was illuminated with 425–445 nm light, and IRIS-2.3_TMR_ was illuminated with 460–490 nm light. Scale bar, 10 μm. (**b**) Dual-FRET imaging of [IP_3_] and [Ca^2+^] in a fertilized mouse egg. Signals from DYC3.60 (ECFP) and IRIS-2.3_TMR_ (EGFP and TMR) from single fertilized mouse egg are shown in the left and right panels, respectively. (**c**) Emission changes in DYC3.60 (black line) and ratio changes of IRIS-2.3_TMR_ (red line) are shown. Sperm was added at time zero. (**d**) Data shown in (**c**) on an enlarged time scale. The arrowheads indicate the time of peaks in DYC3.60 signal (black) and IRIS-2.3_TMR_ signal (red). (**e**) A histogram of the peak time difference between DYC3.60 signals and IRIS-2.3_TMR_ signals (n = 61). The positive value indicates that the peak of DYC3.60 signals precedes that of IRIS-2.3_TMR_ signals (17 ± 11 sec). (**f**) Emission ratio changes of DYC3.60 (black line) and IRIS-2.3_TMR_ (red line) in an unfertilized egg stimulated with 100 μM carbachole. Asterisks show the peak of each Ca^2+^ spike. Carbachole was added during the time indicated by the horizontal bar. (**g**) Data shown in (**f**) on an enlarged time scale around the rise of first Ca^2+^ spike. The dashed lines with red and black triangles indicate the time of peaks in IRIS-2.3_TMR_ signal and DYC3.60 signal, respectively.

We also test our dual-FRET pair for [IP_3_] and [Ca^2+^] at fertilization of mouse eggs. We microinjected cRNAs of DYC3.60 and IRIS-2.3 into the eggs and stained the eggs with TMR. As shown in Fig. 5a, these fluorescent probes were distributed evenly in the egg. Well separation of excitation and emission spectra of these fluorophores enabled simultaneous detection of these fluorescence (Figs 1e, 5a,b). As same as the results we obtained with the pair of Indo-5F and IRIS-2.3_TMR_, we successfully detected fertilization-induced [Ca^2+^] and [IP_3_] changes with DYC3.60 and IRIS-2.3_TMR_ (Fig. 5b and Supplementary video 1). As same as HeLa cells, [IP_3_] at the termination was higher than that at the initiation of Ca^2+^ oscillations in fertilized mouse eggs (Fig. 2i and Supplementary Fig. 5).

### Ca^2+^-induced regenerative IP_3_ production

We also detected delays in the peak of [IP_3_] compared to the peak of each Ca^2+^ spike (17 ± 11 sec, n = 63, Fig. 5c,d), suggesting that Ca^2+^-induced regenerative IP_3_ production through PLC produces small [IP_3_] rises at each Ca^2+^ spike to maintain [IP_3_] over the basal level, which results in long lasting Ca^2+^ oscillations in fertilized eggs. Which PLC isoforms contribute to this regenerative process? Eight PLC isoforms are known to express in the mouse egg: PLCβ1^37^, PLCβ3^37^, PLCβ4^23^, PLCγ1^37^, PLCγ2^37^, PLCδ1^23^, PLCδ4^23^, and PLCε^23^. From these isoforms, knockout mice of PLCβ4, PLCδ1, PLCδ4, and PLCε are born normally^23,38–40^. On the other hand, knockout mice of PLCβ1^41^, PLCβ3^42^, PLCγ1^38^, and PLCγ2^43^ have problems on development of the embryo. However, dominant-negative experiments employing recombinant SH2 domain to inhibit PLCγ1 and γ2 did not inhibit the Ca^2+^ oscillatory pattern during fertilization^44^. Based on these findings, Igarashi *et al*. found that reduced expression of PLCβ1 by RNAi resulted in a significant decrease in Ca^2+^ transients and overexpression of PLCβ1 by cRNA injection resulted in perturbed duration and frequency of Ca^2+^ oscillations^23^. Thus, Ca^2+^ induced activation of PLCβ isozymes are the strong candidates which play a pivotal role to the accelerated production of IP_3_ during Ca^2+^ spikes in fertilized mouse eggs. To determine the role of PLCβ in the mouse egg, we stimulate unfertilized mouse eggs with 100 μM carbachole (Fig. 5f,g). The stimulation caused Ca^2+^ spikes and a monotonic [IP_3_] rise (Fig. 5f). At fertilization, [IP_3_] changes always follow after Ca^2+^ spikes. On the other hand, Ca^2+^ spikes did not accompany with delayed [IP_3_] rises in carbachole stimulated unfertilized eggs (Fig. 5f). Particularly, IP_3_ peak at the first Ca^2+^ spike preceded Ca^2+^ peak (Fig. 5g). These data showed that Ca^2+^-induced IP_3_ producing activity is not strong in unfertilized eggs, suggesting that sperm derived PLCζ should participate Ca^2+^-induced IP_3_ production. As we showed in Figs 3a,b and 4, Ca^2+^-induced [IP_3_] rises increased later phase of fertilization-induced Ca^2+^ oscillations, suggesting that fertilization induces quantitative or qualitative changes of PLC in later phase of Ca^2+^ oscillations.

## Discussion

In this study, we developed a dual-FRET pair of biosensors for the detection of [IP_3_] and [Ca^2+^] in mammalian cells. The uniqueness of our dual-FRET pair is using single fluorophore for one of the pairs. Replacement of Y145 to W in EYFP is known to produce a non-fluorescent chromoprotein that retains its absorption of emission light^26^. Introduction of the Y145W mutation into cp173Venus of YC3.60^27^ resulted to produce single fluorophore with fluorescent quencher in the Ca^2+^ FRET sensor, DYC3.60. Usually, four fluorophores are necessary for dual-FRET imaging. Most of FRET sensors have cyan and yellow fluorescent proteins^45^, and these fluorescent proteins cover a broad spectral profile. Thus, using FRET sensor with cyan and yellow proteins, it is difficult to find a partner FRET sensor for dual-FRET imaging without using spectral unmixing to distinguish each fluorescent signal mathematically from significantly overlapped fluorescent signals^46,47^. We offer a dual-FRET imaging with three fluorophores, which gives easier detection and separation of fluorescent signals.

The new FRET sensors enabled imaging of [IP_3_] and [Ca^2+^] at fertilization of mouse eggs. We have succeeded to detect [IP_3_] changes in fertilized mouse eggs using a second-generation fluorescent IP_3_ sensor, IRIS-2.3, which has an improved dynamic range and a high IP_3_ sensitivity. Simultaneous monitoring of both Ca^2+^ and IP_3_ in fertilized mouse eggs showed that the [IP_3_] increase was detected approximately 3 min before the onset of the first Ca^2+^ transient. The result is consistent with the expectation that highly Ca^2+^ sensitive PLCζ produces IP_3_ at the basal level of [Ca^2+^] in the egg cytosol after sperm-egg fusion^12^. Mehlmann and Kline reported microinjection of small amount of IP_3_ (8.6 nM) is able to induce Ca^2+^ spike in unfertilized mouse eggs^48^. Our measurements showed the same results that the amount of IP_3_ produced in mouse eggs is small even after the fertilization because only IRIS-2.3, which shows the highest IP_3_ sensitivity (Kd = 47 nM) among the IP_3_ sensors developed, could detect [IP_3_] increases at the onset of the first Ca^2+^ transients.

IP_3_R has a bell-shaped calcium response curve: the open probability of IP_3_R is activated by low [Ca^2+^] and inhibited by high [Ca^2+^]^32^. Based on this finding, De Young and Keizer reported a mathematical model to reproduce Ca^2+^ oscillations with constant [IP_3_]^24^. In this and previous reports, we showed sustained [IP_3_] increase during Ca^2+^ oscillations in HeLa cells and fertilized mouse eggs^25^ (Figs 3 and 4), and the same results were obtained with other IP_3_ sensor proteins^29,30^. Consistently with our results, Mehlmann and Kline reported single microinjection of IP_3_ induces Ca^2+^ oscillations in unfertilized mouse eggs^48^. Jones *et al*. also reported Ca^2+^ oscillations with continuous low level caged-IP_3_ photolysis in unfertilized mouse eggs^49^. PLCζ is a smallest and simplest PLC isoform^9^. The activity of PLCζ is regulated by Ca^2+^ and localization into nucleus after pronuclear formation, and other regulations are not known^50^. PLCζ has highest Ca^2+^ sensitivity compared to the other PLC isoforms and is 70% active at the basal level [Ca^2+^] in cells^12^. Thus, PLCζ should be continuously active after fertilization until pronuclear formation^51^, which should sustain continuous [IP_3_] increase during fertilization-induced Ca^2+^ oscillations (Figs 3 and 4). We previously found that CICR dominantly work as a positive feedback loop to produce the rising phase of Ca^2+^ spikes in HeLa cells^25^. Our data suggest that the mechanism elicits the rising phase of Ca^2+^ spikes in fertilized mouse eggs is more complex. Initially, CICR dominantly works as the positive feedback loop, and Ca^2+^-induced IP_3_ production gradually participates to produce Ca^2+^ spikes cooperatively with CICR in the later phase of Ca^2+^ oscillations. Ca^2+^-induced IP_3_ production through PLC produces [IP_3_] rises at each Ca^2+^ spike to help keeping [IP_3_] over the basal level, which results in long lasting Ca^2+^ oscillations in fertilized eggs.

In conclusion, we produced FRET sensors with new choices of fluorophores for dual-FRET imaging of [IP_3_] and [Ca^2+^]. Less overlaps of excitation and emission spectrum of IRIS-2s and DYC3.60 allowed dual-FRET imaging of Ca^2+^ and IP_3_ even without spectral unmixing. Because of the smaller number of fluorophores, our dual-FRET approach can reduce the effort to detect each fluorescent signal separately. The wider dynamic range and higher sensitivity achieved by IRIS-2.3 will enable the detection of subtle [IP_3_] changes associated with [Ca^2+^] changes at egg fertilization to local [Ca^2+^] increase events.

## Materials and Methods

### Animals

Experiments used ddY mice for preparation of oocytes and sperm. All animal experiments were performed in accordance with the guidelines approved by the Animal Experiments Committee of RIKEN Brain Science Institute. All experiments were carried out in accordance with the approved ethical guidelines and regulations.

### Gene construction

The FRET donor and acceptor of IRIS-1 were replaced with mEGFP and Halo-protein (Promega), respectively, to produce IRIS-2. Amino acid residues 224–575 of mouse IP3R1 in IRIS-2 were replaced with amino acid residues 224–579 of mouse IP3R1 to produce IRIS-2.3. The Y145W mutant^26^ of circular permutated Venus (cp173V-Y145W)^27^ was generated using the site-directed mutagenesis. The FRET acceptor of YC3.60^27^ was replaced with cp173Venus-Y145W to produce DYC3.60. IRIS-2, IRIS-2.3 and DYC3.60 cDNAs were cloned into the NheI and XbaI sites of pcDNA3.1 zeo(+) (Invitrogen) for the expression in HeLa cells. The cDNAs were cloned into the XbaI site of pTNTTM (Promega) with extended poly(A) tail (57 residues) and synthesized cRNAs were injected into mouse oocytes.

### Protein expression and purification

The full-length cDNA of IRIS-2 was isolated from pcDNA3.1 zeo(+)-IRIS-2 by using NheI and XbaI sites and was cloned into the XbaI site of baculovirus transfer vector pFastBac1 (Invitrogen). The recombinant baculovirus was used for the large-scale expression of IRIS-2 in Sf9 cells as described previously^52^. The expressed proteins were purified on a HiTrap heparin HP column (GE Healthcare Life Sciences) as described previously^53^.

### Cell culture and transfection

HeLa cells were cultured in Dulbecco’s modified Eagle medium supplemented with 10% heat-inactivated fetal bovine serum. HeLa cells were transfected with expression vectors by transfection reagent (Mirus TransIT). One day after the transfection, cells were used for imaging experiments.

### Preparation of RNA

Plasmids carrying IRISs or DYC3.60 were digested by NdeI, and linearized DNA fragments were purified with Wizard SV Gel and PCR clean-up Kit (Promega). They were used as the templates for RNA transcription by T7 polymerase using T7 mMESSAGEmMACHINE Kit (Ambion). RNA was purified using RNeasy MinElute Cleanup Kit (Qiagen) and stored at −80 °C until use.

### Preparation of gametes

Full grown immature oocytes were collected from the follicles in the ovaries of female mice 47–49 h after the injection of pregnant mare serum gonadotropin. Isolated oocytes were freed from cumulus cells mechanically by pipetting in M2 medium, and then cRNAs of IRIS-1, IRIS-2, IRIS-2.3, DYC3.60 or dKeima570 were injected as described below. Sperm was collected from the caudal epididymides and were incubated in M16 medium^54^ supplemented with 4 mg/ml BSA (Sigma) at 37 °C (5% CO2) for >5 h for capacitation and acrosome reaction^55^.

### Microinjection and insemination

RNA solutions were diluted to 130 ng/μl with the intracellular medium (150 mM KCl, 5 mM Tris-KOH, pH 7.0). Immature oocytes were injected with 20 pl of RNA solutions and incubated in the M16 medium for 16 h at 37 °C with 5% CO_2_. Only eggs maturated normally to metaphase II with the first polar body were used in the following experiments. After loaded with 2 μM of Indo-5F or Fura-2 for 30 min in the M2 medium, eggs were freed from the zona pellucida by brief treatment with acidic Tyrode’s solution (pH 2.5)^56^ for insemination. Sperm was added during imaging experiments.

### Imaging

After loading HeLa cells with 10 μM Indo-5F-AM (AnaSpec), imaging was performed under the constant flow (2 ml/min) of the balanced salt solution containing 20 mM Hepes, pH 7.4, 115 mM NaCl, 5.4 mM KCl, 1 mM MgCl2, 1.3 mM CaCl2, and 10 mM glucose as an imaging media at 37 °C through an inverted microscope (IX71 or IX81; Olympus) with a cooled charge-coupled device (CCD) camera (ORCA-ER; Hamamatsu Photonics) and a 40x, 1.35 NA, oil-immersion objective (Olympus). For the fluorescent images of IRIS-1 and Indo-5F, an emission splitter (W-view; Hamamatsu Photonics) was used with a light source exchanger (DG-4; Sutter Instrument Co.) on the IX71 inverted microscope. Sequential excitation of IRIS-1 and Indo-5F was performed by using a 450-nm dichroic mirror and two excitation filters (a 425–445 nm filter for IRIS-1 and a 360-nm filter for Indo-5F). Emissions from IRIS-1 and Indo-5F were split with a 460–510-nm filter (for IRIS-1 and Indo-5F), a long-path 520-nm (for IRIS-1) barrier filter, and two 505-nm dichroic mirrors equipped in W-view.

Eggs were incubated with M2 buffer at 37 °C on IX81 inverted microscope. Ca^2+^ and IP_3_ were visualized with sets of Indo-5F and IRIS-1, Indo-5F and IRIS-2, Indo-5F and IRIS-2.3, Fura-2 and IRIS-2.3, or DYC3.60 and IRIS-2.3, respectively. Sequential excitation of Ca^2+^ and IP_3_ indicators was performed by using dichroic mirrors (a 400-nm mirror for Indo-5F and a 450-nm mirror for IRIS-1 and DYC3.60 and a 505-nm mirror for IRIS-2 and IRIS-2.3) and excitation filters (a set of 340 and 380-nm filters for Fura-2 and a 380-nm filter for Indo-5F and a 425–445 nm filter for IRIS-1 and DYC3.60 and a 460–490-nm filter for IRIS-2 and IRIS-2.3). Emissions from Ca^2+^ and IP_3_ indicators were split with emission filters (a set of 400–420 and 460–510-nm filters for Indo-5F and a set of 460–510 and 525–565 filters for IRIS-1 and DYC3.60 and a 510–550 filter for Fura-2 and a set of 510–550 and 573–613-nm filters for IRIS-2 and IRIS-2.3_TMR_), and three filter exchangers (Lamda 10; Sutter Instruments, IX2-RFACA; Olympus).

Image acquisition was performed with MetaFluor (Molecular Devices). Data analysis was performed with MetaFluor and Igor Pro (WaveMetrics) softwares. The EGFP/TMR emission ratio (IRIS-2s), the ECFP/Venus emission ratio (IRIS-1s), the dKeima570/ECFP emission ratio (DYC3.60), the 420–440 nm/460–510 nm emission ratio (Indo-1) and the ratio of 510–550 nm emission excited at 340 nm and 510–550 nm emission excited at 380 nm (Fura-2) were defined as R. ∆R was defined as R - Rbase, where Rbase is the basal level of R. Baseline drift in each experiment was corrected with subtracting the trend line which is calculated with the line around the beginning of each experiment.

### Uncaging of caged-IP_3_

HeLa cells transfected with IRIS-2 and DYC3.60 were loaded with 10 μM membrane permeable caged-IP_3_ (iso-Ins(1,4,5)P_3_/PM (caged), Enzo Life Science). The uncaging stimulation was done with extra light source (mercury lamp) equipped in IX81, filtered by a 333–348-nm filter and a 400-nm dichroic mirror, illuminated the cells through 20x, 0.50 NA, water-immersion objective (Olympus).

## Supplementary information

Supplementary Figures

Supplementary Video 1
