# Supplementary material for: Dual-FRET imaging of IP3 and Ca2+ revealed Ca2+-induced IP3 production maintains long lasting Ca2+ oscillations in fertilized mouse eggs
Source: Sci Rep. 2019 Mar 18;9:4829. doi: 10.1038/s41598-019-40931-w (PMC6423007; doi:10.1038/s41598-019-40931-w)
Supplement: Supplementary file 1 — Supplementary Figures [file 41598_2019_40931_MOESM1_ESM.pdf]

Dual-FRET imaging of IP_3 and Ca^{2+} revealed Ca^{2+}-induced IP_3 production maintains long lasting Ca^{2+} oscillations in fertilized mouse eggs

Toru Matsu-ura[1], Hideki Shirakawa[2], Kenichi G.N. Suzuki[3], Akitoshi Miyamoto[4,5], Kotomi Sugiura[6], Takayuki Michikawa[7], Akihiro Kusumi[8], and Katsuhiko Mikoshiba[4*]

Affiliation:

1. Department of Stem Cell Pathology, Kansai Medical University, 2-5-1 Shin-machi, Hirakata, Osaka, 573-1010, Japan.
2. Department of Applied Physics and Chemistry, The University of Electro-Communications, Tokyo 182-8585, Japan
3. Center for Highly Advanced Integration of Nano and Life Sciences (G-CHAIN), Gifu University, 1-1 Yanagido, Gifu 501-1193, Japan
4. Laboratory for Developmental Neurobiology, Center for Brain Science, RIKEN, 2-1 Hirosawa, Wako, Saitama 351-0198, Japan
5. Laboratory of Single-Molecule Cell Biology, Kyoto University Graduate School of Biostudies, Konoe-cho, Sakyo-ku, Kyoto 606-8501, Japan
6. Division of Mucosal Vaccines, International Research and Development Center for Mucosal Vaccine, The Institute of Medical Science, University of Tokyo, 4-6-1 Shirokanedai, Minato-ku, Tokyo 108-8639, Japan
7. Laboratory for Biotechnological Optics Research, Center for Advanced Photonics, RIKEN, 2-1 Hirosawa, Wako, Saitama 351-0198, Japan
8. Okinawa Institute of Science and Technology Graduate University, 1919-1 Tancha, Onna, Okinawa, 904-0412, Japan

[#]**Corresponding to:** Katsuhiko Mikoshiba

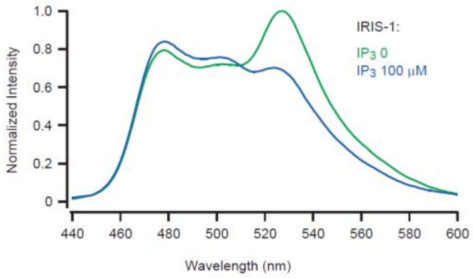

Supplementary Figure 1. Emission spectra of IRIS-1. Spectrum measurements of purified IRIS-1 were obtained with (blue line) and without (green line) addition of IP_3.

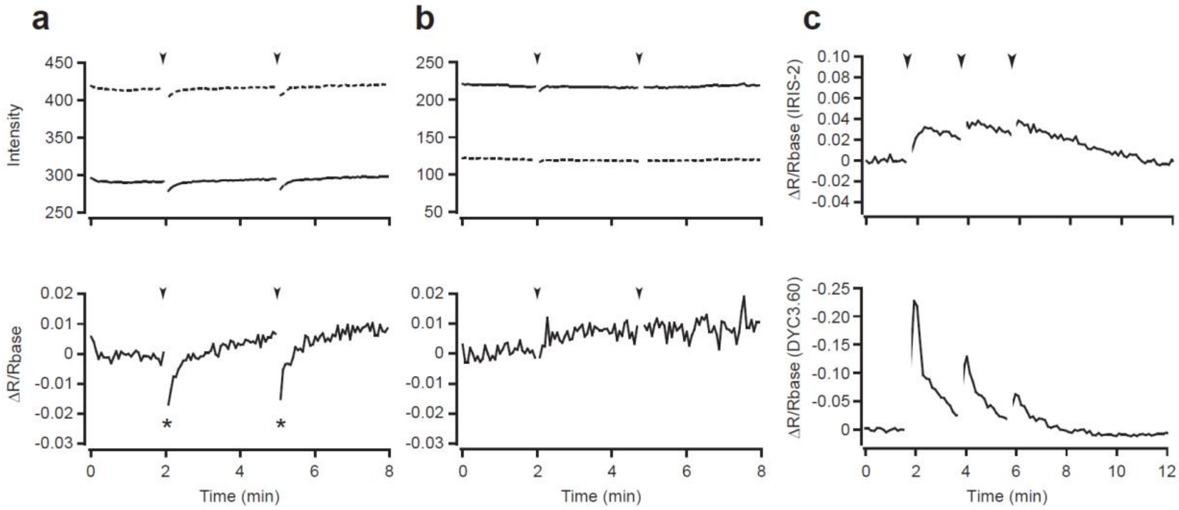

Supplementary Figure 2. Uncaged-IP_3 was detected by IRIS-2$_{TMR}$ expressed in HeLa cells. (a and b) The effect of UV on the fluorescence of IRIS-1 (a) and IRIS-2$_{TMR}$ (b). Arrow heads indicate the timing of UV irradiations. Upper panels show fluorescent signals of FRET donors (continuous lines; ECFP and EGFP in a and b, respectively) and acceptors (broken lines; Venus and TMR in a and b, respectively) in HeLa cells. Lower panels show FRET ratio of IRIS-1 (a) and IRIS-2 (b). (c) IRIS-2$_{TMR}$ detected UV-uncaged IP_3 which induce Ca^{2+} release from IP_3R. Fluorescent ratio of IRIS-2$_{TMR}$ (upper panel) and DYC3.60 (lower panel) are plotted.

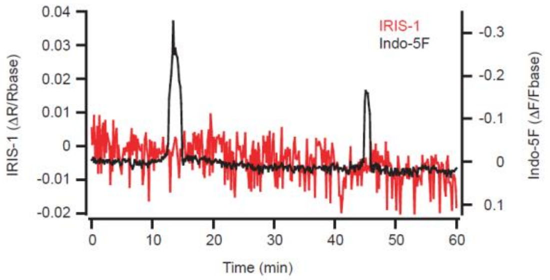

Supplementary Figure 3. Fluorescent ratio changes of IRIS-1 during fertilization in mouse eggs. Signals of IRIS-1 are plotted with red lines. Fertilization induced Ca^{2+} spikes were detected by Indo-5F (black lines). Sperm was added at the time of zero. Fluorescent images were acquired each 4 sec.

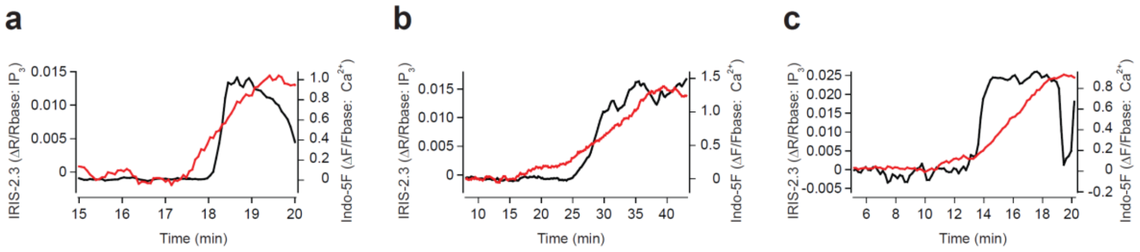

Supplementary Figure 4. [IP$_3$] and [Ca^{2+}] traces obtained with Indo-5F and IRIS-2.3$_{TMR}$ at the onset of first Ca^{2+} spike. (a-c) Three independent imaging experimental results are shown. [IP$_3$] (red lines) and [Ca^{2+}] traces (black lines) were obtained with IRIS-2.3$_{TMR}$ and DYC3.60, respectively.

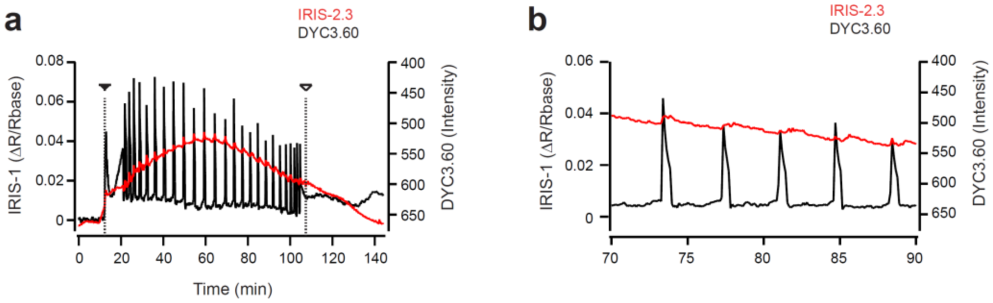

Supplementary Figure 5. Dual FRET imaging of whole Ca^{2+} oscillations at fertilization. (**a**) Emission changes of DYC3.60 (black line) and ratio changes of IRIS-2.3$_{TMR}$ (red line) are shown. Initiation of the first Ca^{2+} spike and the termination of Ca^{2+} oscillations were marked with dashed line with closed and open triangles, respectively. Sperm was added at time zero. (**b**) The enlarged graph of **a** shows delays in [IP_3] rises.
